# Observations on the Management and Preservation of the Eyes

**Published:** 1805-07-01

**Authors:** 

**Affiliations:** Newman Street, Oxford Road


### Observations on the Management and Preservation of the Eyes. *Communicated by* Dr. Lang, *of Newman Street, Oxford Road.*

In a Treatise on the Disorders of the Eyes, which I read lately, there was mentioned *obiter*, that if luminous rays fall on the pupil of one eye only, that of the other, which is not at all exposed to them, will however contract itself in some degree, though not quite so much as the former. This put me in mind of some remarks I had heard of the celebrated Dr. Schimdt, at Vienna, when I attended his practical institution, shewing at the same time, that they were not so universally known as I should have imagined them to be. As they are of some practical moment, I venture to offer them to you for insertion in your valuable publication, if you should think them worthy of a place therein.

Dr. Schmidt, like the author of the above mentioned Treatise (the Title of which I am not able to recollect) observed at first, only the fact I have cited; but upon further investigation he found, that the reverse of it is equally certain to happen under the contrary circumstances, viz. if you deprive one eye of the influence of luminous rays, and by that means dilate its pupil, that of the other eye, which is still under the effects of light, will dilate itself likewise in some degree, and in proportion to the dilatation of the former. These two facts point out more clearly the sympathy and close connection of our organs of sight, and in my opinion, contribute very much to evince the position, that in the *chiasma nervorum opticorum*, the substance of the optical nerves is really and effectually incorporated together, and that those nerves do not decussate one over or through the other. As this, however, is more related to speculation than to any thing else, I shall not enter into the subject, but proceed to other conclusions to be drawn from the stated facts, concerning the management and preservation of our eyes.

It is universally known, that the pupils become contracted in proportion to the light falling on their *retinas*; and if there are many rays acting on the retina, the pupil will contract itself, until so many only are permitted to pass as are sufficient to form a distinct image of the object we behold, without dazzling the eye. On the contrary, the pupil dilates itself in the dark to a great degree, to permit every atom of light to make its way to the *retina*. Now in using a single perspective, or eye-glass, the generality generality of people are in the habit of applying it to the same eye, and at the same time of shutting the other. According to Dr. Schmidt's observations, the necessary consequence must be, that the pupil of that eye, to which the glass is applied, is considerably more enlarged than it ought to be in proportion of the light acting upon its *retina*, because of the enlargement of the pupil of the other eye, which is shut, and consequently in the dark, occasioning the pupil of the open eye to dilate itself. This accounts partly for the uneasiness we feel, when we have for some time looked through a glass with one eye shut; though the indistinct image we receive through the means of glasses has likewise a share in it, and that uneasiness in a convex eye can therefore never be entirely obviated. To avert, at least, as many ill consequences as possible from the use of perspectives, we should not only employ *achromatic* glasses, but, for the sake of this sympathic enlargement of the pupils, we ought to make use of a double perspective, or *binoculum*, as the opticians call it; and if such a one is not to be procured, we should always keep the other eye at least *half* open. The reason why it must not be quite opened, is, because the other eye, with which we look through the glass, would, on account of the sympathetic contraction of the pupils, not receive so much light as that which is quite uncovered, and of course we should be subject to the contrary disadvantage.

For the purpose of having more light, and in order not to have this light overshadowed by our own hand, we commonly place our writing desks with the left side to the window, or to the light in general, as most people write with the right hand. But this produces, though in a less degree, the same consequences as the common use of perspectives; for although the rays, reflected from that material on which we write, will almost equally affect both our eyes, yet those additional rays, which we receive through the window, or from a luminous body at our left side, can only affect our left eye, as the ridge of the nose will hinder them from entering our right eye. Hence the pupil of the latter must be more contracted, as it should be, by the rays it receives, and accordingly we endeavour (though imperceptibly to ourselves, from want of attention) to adjust that eye better and better to the smaller quantity of light it receives, in opposition to the re-action of the left eye. The consequences of this are easily to be drawn; if both eyes are sound, the sight of the left will by and by be far inferior to that of the right, because this latter latter is more in the habit to be closer employed, and it is, by the like diameter of the pupil, easier to be affected from the same quantity of light than the former, which is accustomed to a greater quantity of it; if, on the contrary, the eyes are naturally weak, they will both become in a much less time short-sighted than in a better situation, as the unequal affection of them will mutually and alternately contribute to accelerate that event. To prevent this, it will be adviseable to place ourselves in writing, or any similar occupation, with our face *before* a window, that we may have the light in front; and if we are obliged to make use of artificial light, we must place it likewise in front of ourselves, and effect, by means of a screen of nearly transparent paper, its equal distribution before our eyes.

It may also be observed here, that we do not, by far, take sufficient care of our eyes with respect to the manner of lighting our apartments, and especially, when we are writing, reading, &c. In the first place, we do not give light enough, which induces us, secondly, to have our candles so low, that the flame of them is constantly fluttering before our eyes. It is evident, that no one of the objects around us can give so bright a light as the candle itself, as they all borrow their light from it; consequently, if we have a candle before us, whose flame is not above our horizon, and we look at another object, not only the luminous rays refracted from that, but also a quantity of rays, emanating from the candle, must touch our eyes. The pupil, therefore, will be much more contracted, as it ought to be, by virtue of the *refracted rays*, which produce an indistinct and imperfect image of the object we are really looking at. But this indistinctness generates not only an uneasiness in our mind, but we also endeavour to correct it, by pinching our eyes with the eyelids, retracting and protruding them, and, in short, making various trials to effect a distinct image, when the organ is soon tired out, and we are obliged to let it rest. Here every body exclaims, it is the easiest matter in the world to avoid this, if you only place a green shade over your eyes. But, however, there is another preventive, which is, in my opinion, still easier, and more efficacious, without having the disadvantages of a shade, namely, if we augment the light, and elevate it so much, that it is above our horizon. It is true, the shade obstructs the rays of the candle, but then we shall frequently be obliged to turn from our work, when the light of the candle will, on a sudden, start in our eyes; and every one knows, how very hurtful sudden changes changes of a weak and strong light are to the sight. Moreover, if the light is very low, it will be concentrated almost upon one spot; and as the ground on which we work is generally of a white colour (as paper, linen, &c.) the strong reflection will dazzle our eyes so much, that we must take considerable pains to observe the very work we are doing. If it was not for the high price, I should recommend for the above desired purposes *Argand's* lamps, which unite every conveniency in themselves, if they are suspended high enough, as they give a sufficiently strong and equal light, being at the same time sufficiently above the horizon of our eyes.

*June* 9, 1805.

